# 2′-Fucosyllactose inhibits human norovirus replication in human intestinal enteroids

**DOI:** 10.1128/jvi.00938-24

**Published:** 2025-01-10

**Authors:** Ketki Patil, B. Vijayalakshmi Ayyar, Nicole M. Hayes, Frederick H. Neill, Lars Bode, Mary K. Estes, Robert L. Atmar, Sasirekha Ramani

**Affiliations:** 1 Department of Molecular Virology and Microbiology, Baylor College of Medicine 189531 , Houston, Texas, USA; 2 Department of Pediatrics, Larsson-Rosenquist Foundation Mother-Milk-Infant Center of Research Excellence (MOMI CORE), and the Human Milk Institute (HMI), University of California San Diego 547075 , La Jolla, California, USA; 3 Department of Medicine, Baylor College of Medicine 171841 , Houston, Texas, USA; University of North Carolina at Chapel Hill, Chapel Hill, North Carolina, USA

**Keywords:** norovirus, human milk oligosaccharide, 2’-fucosyllactose, enteroids, antiviral, therapeutic

## Abstract

Human noroviruses (HuNoVs) are the leading cause of acute gastroenteritis worldwide. Currently, there are no targeted antivirals for the treatment of HuNoV infection. Histo-blood group antigens (HBGAs) on the intestinal epithelium are cellular attachment factors for HuNoVs; molecules that block the binding of HuNoVs to HBGAs thus have the potential to be developed as antivirals. Human milk oligosaccharides (HMOs) are glycans in human milk with structures analogous to HBGAs. HMOs have been shown to act as decoy receptors to prevent the attachment of multiple enteric pathogens to host cells. Previous X-ray crystallography studies have demonstrated the binding of HMO 2′-fucosyllactose (2′FL) in the same pocket as HBGAs for some HuNoV strains. We evaluated the effect of 2′FL on the replication of a globally dominant GII.4 Sydney [P16] HuNoV strain using human intestinal enteroids (HIEs) from adults and children. A significant reduction in GII.4 Sydney [P16] replication was seen in duodenal and jejunal HIEs from multiple adult donors, all segments of the small intestine from an adult organ donor, and in two pediatric duodenal HIEs. However, 2′FL did not inhibit HuNoV replication in two infant jejunal HIEs that had significantly lower expression of α1–2-fucosylated glycans. 2′FL can be synthesized in large scale, and safety and tolerance have been assessed previously. Our data suggest that 2′FL has the potential to be developed as a therapeutic for HuNoV gastroenteritis.

**IMPORTANCE:**

Human noroviruses infect the gastrointestinal tract and are a leading cause of acute gastroenteritis worldwide. Common symptoms of norovirus include diarrhea, vomiting, and stomach cramps. Virus shedding and symptoms are prolonged and debilitating in immunocompromised patients. Currently, there are no approved vaccines or targeted antivirals for treating human norovirus infection. Human intestinal enteroids derived from intestinal stem cells allow the successful replication of norovirus in the laboratory and can be used as a physiologically relevant model system to evaluate antivirals. We discovered that 2′-fucosyllactose (2′FL), an oligosaccharide naturally occurring in human milk, inhibits GII.4 norovirus replication in HIEs from multiple donors and thus has the potential to be developed as a therapeutic for human norovirus. These findings have high translational potential since 2′FL from several manufacturers has a “generally recognized as safe” status and can be synthesized on a large scale for immediate application.

## INTRODUCTION

Human noroviruses (HuNoVs) are a leading cause of acute gastroenteritis across all age groups (1). There are an estimated 677 million HuNoV infections worldwide and over 200,000 HuNoV-associated deaths each year, with the latter mainly reported in low- and middle-income countries (2, 3). HuNoV outbreaks have been reported in hospitals, long-term care facilities, cruise ships, planes, and restaurants (4). Each year, HuNoV infections can result in more than $4 billion and $60 billion in direct healthcare and societal costs, respectively (5). Currently, there are no targeted antivirals or licensed vaccines for HuNoVs.

Host cellular factors involved in virus attachment and entry are potential targets for antiviral development. Histo-blood group antigens (HBGAs) are cellular attachment factors for HuNoVs (6). These complex carbohydrates are present on red blood cells, mucosal epithelial cells, and biological fluids (7). Human milk contains a group of structurally diverse unconjugated glycans, with some structures analogous to HBGAs (8). These sugars, called human milk oligosaccharides (HMOs), comprise 5–15 g/L of mature human milk and are the third most solid abundant component of human milk after lactose and lipids (9, 10). More than 150 HMO structures have been identified (11). In addition to serving as prebiotics for bacteria in the infant gut, other functions of HMOs include modulating epithelial and immune cell responses and acting as decoy receptors to reduce the attachment of pathogenic microbes to cell surface receptors (12). As such, HMOs have been shown to prevent pathogen adhesion to host epithelia for multiple enteric bacteria, such as *Campylobacter jejuni*, *Clostridioides difficile*, and *Escherichia coli* O157, as well as viruses, such as rotavirus, coxsackievirus A9, and severe acute respiratory syndrome coronavirus 2 (13–18).

Previous X-ray crystallography studies with several HuNoV genotypes (GI.1, GII.4, GII.10, and GII.17) have shown that 2′-fucosyllactose (2′FL), an α1–2-fucosylated HMO, binds to the protruding domain of the HuNoV capsid protein VP1 in a similar pocket as HBGAs (19–22). 2′FL has also been found to block the binding of HuNoV virus-like particles (VLPs) to porcine gastric mucin (PGM) and saliva that contains HBGAs (19, 21, 22). These data suggest that 2′FL can potentially act as a decoy receptor for HuNoVs. We previously standardized a pipeline to evaluate antivirals against HuNoVs in human intestinal enteroids (HIEs) (23). In the present study, we used this pipeline to evaluate the effect of 2′FL on the replication of GII.4 Sydney [P16] HuNoV and demonstrate a significant reduction in HIEs from multiple donors and intestinal segments.

## RESULTS

### 2′FL significantly reduces GII.4 VLP binding to PGM

**Fig 1:**
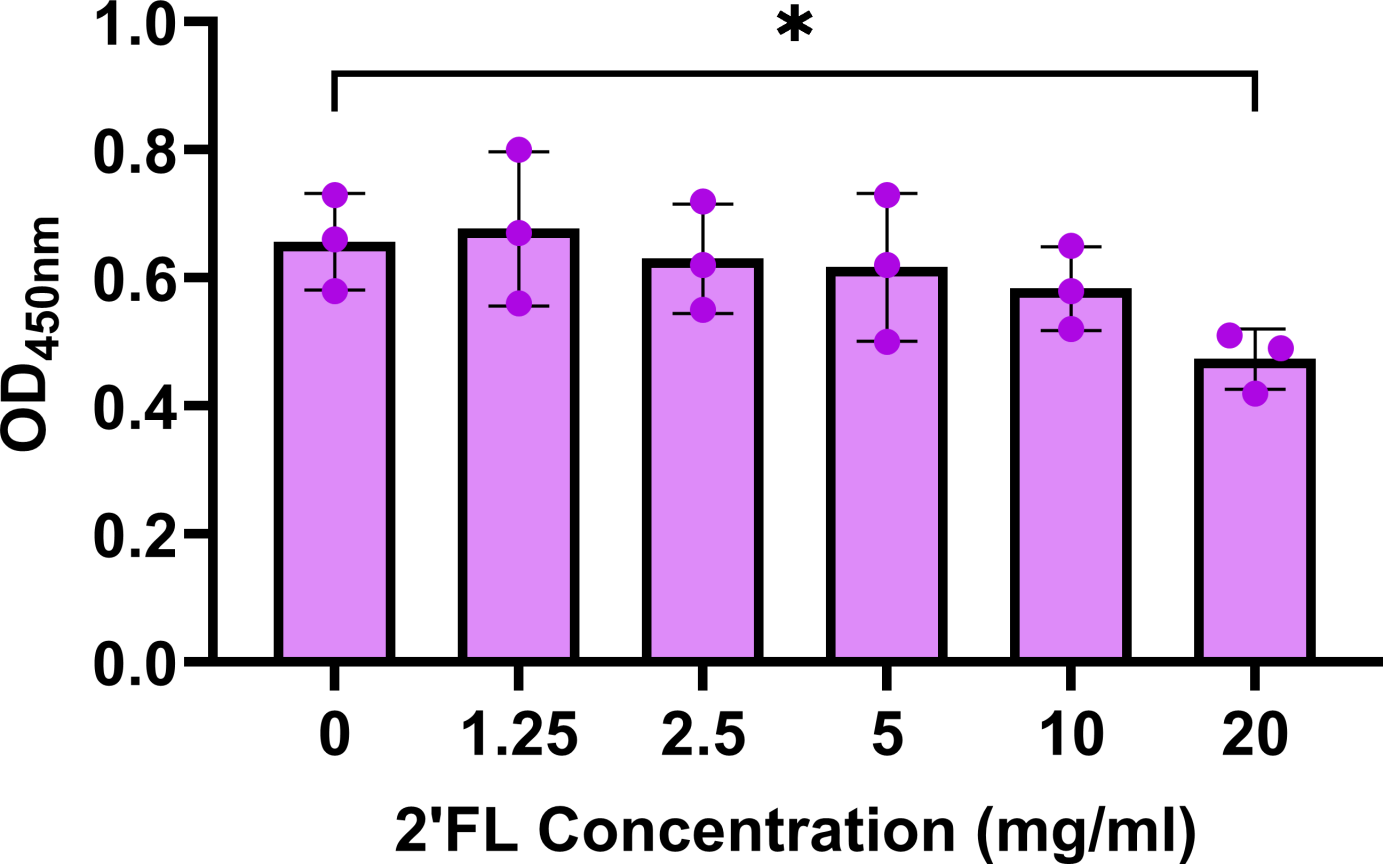
20 mg/mL 2′FL significantly reduces GII.4 Sydney 2012 VLP binding to PGM. Dose–response studies were carried out testing 1.25, 2.5, 5, 10, and 20 mg/mL of 2′FL with 2.5 ug/mL VLPs. All comparisons were made to the condition where 2′FL was not present (0 mg/mL). Data (mean ± standard deviation) represented are from *n* = 3 independent experiments with averages from three technical replicates per experiment. The P-values were calculated using Student’s *t*-test. *P ≤ 0.05.

We first carried out dose–response assays using different concentrations of 2′FL (1.25, 2.5, 5, 10, and 20 mg/mL) to determine if this HMO can reduce the binding of GII.4 Sydney 2012 VLPs to PGM. There was a dose-dependent reduction in VLP binding to PGM, with a significant reduction at 20 mg/mL of 2′FL (Fig. 1), suggesting that 2′FL can potentially act as a decoy to block HuNoV replication.

### Human norovirus tissue culture infectious dose differs per HIE line

We demonstrated previously that the number of genome equivalents (GE) per 50% tissue culture infectious dose (TCID_50_) of HuNoV strains differs in each HIE line (23). Since we planned to examine the effect of 2′FL in inhibiting HuNoV replication using HIE lines from different ages and intestinal segments, we determined the GE/TCID_50_ of the GII.4 Sydney [P16] HuNoV strain in each line to standardize the amount of infectious virus used across HIE lines. The average GE/TCID_50_ from two independent experiments are reported in Table 1, with adult duodenal HIEs requiring the highest number of GE/TCID_50_. For the duodenum and jejunum where HIEs from adults and children were available, the GE/TCID_50_ was lower in HIE lines from children. For HIEs derived from different intestinal segments of the same donor, the highest GE/TCID_50_ was seen in the duodenal HIE D2004, while the ileal line I2004 had lower GE/TCID_50_ values similar to those of infant jejunal lines (J1005 and J1006). Taken together, these data indicate segment- and age-specific differences in GE/TCID_50_ and the need to standardize the inoculum used in infectivity assays to allow for comparison of results between HIE lines.

**TABLE 1:** Summary of genome equivalents (GE) per 50% tissue culture infectious dose (TCID_50_)

HIE	Segment	Age	GE/TCID_50_ ± SD
D109	Duodenal	44 years	4.35 ± 0.28
D2004	Duodenal	25 years	4.54 ± 0.17
J2	Jejunal	52 years	4.05 ± 0.00
J11	Jejunal	52 years	4.06 ± 0.15
J2004	Jejunal	25 years	4.30 ± 0.03
I2004	Ileal	25 years	3.76 ± 0.06
4D	Duodenal	2 years	4.23 ± 0.23
8D	Duodenal	5 years	3.84 ± 0.21
J1005	Jejunal	10 weeks	3.78 ± 0.25
J1006	Jejunal	12 weeks	3.63 ± 0.06

^*a*^ List of HIE lines, in which the segment of origin and age of donors are shown. GE/TCID50 values are shown as log_10_ values ± standard deviation (SD) from *n* = 2 independent experiments.

### 2′FL significantly reduces GII.4 human norovirus replication in adult HIE lines

**Fig 2:**
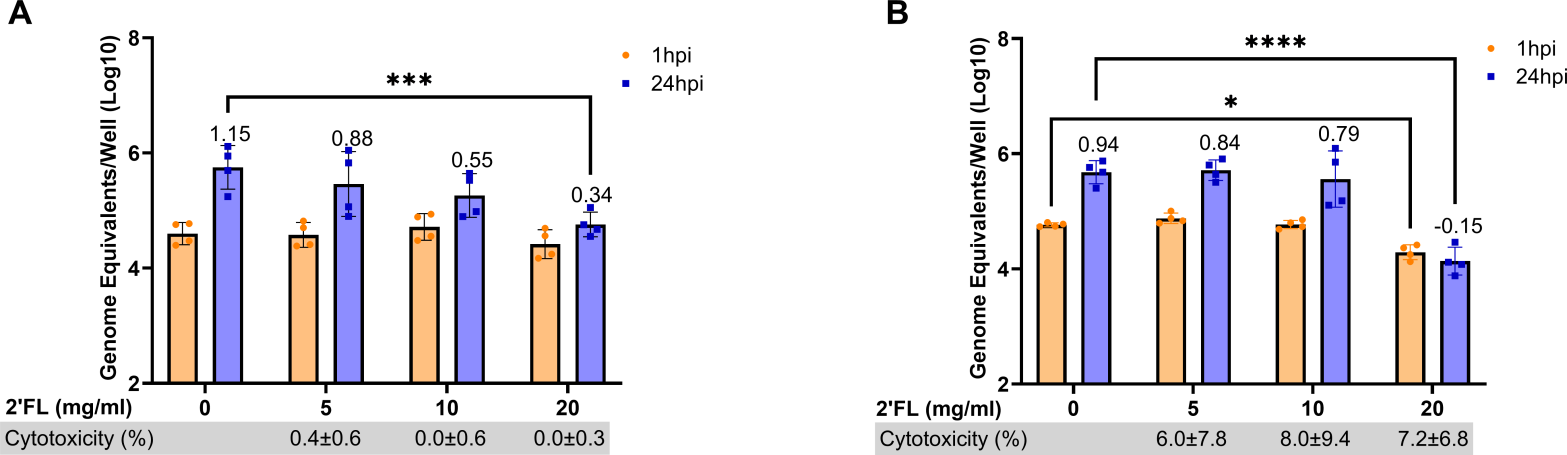
20 mg/mL 2′FL significantly reduces GII.4 Sydney [P16] HuNoV replication in adult duodenal HIE lines. Dose–response assays were carried out in adult duodenal HIE lines (**A**) D109 and (**B**) D2004 using 5, 10, and 20 mg/mL of 2′FL. GE/well was determined by RT-qPCR at 1 h post-infection (hpi) and 24 hpi. Numbers above the bars indicate log_10_ fold change comparing GE/well at 24 to 1 hpi. Cytotoxicity (measured by the lactase dehydrogenase assay) is represented in percentage below each graph. Data represented are means ± standard deviation (SD) from *n* = 2 independent experiments with two technical replicates per experiment. The P-values were calculated using analysis of variance, Sidak’s multiple comparisons test. *P ≤ 0.05, ***P ≤ 0.001, ****P ≤ 0.0001.

We first carried out dose–response assays to determine if 2′FL inhibited GII.4 Sydney [P16] HuNoV binding and replication in HIEs. Although the significant inhibition of VLP binding to PGM was seen only with 20 mg/mL of 2′FL, we tested two additional concentrations (5 and 10 mg/mL) to determine if lower doses could be effective in infectivity studies. HIE lines were infected with 100 TCID_50_ of GII.4 Sydney [P16] HuNoV based on their respective GE/TCID_50_ (Table 1). In the absence of 2′FL, GII.4 Sydney [P16] HuNoV showed ~1log_10_ increase in GE/well at 24 h post-infection (hpi) compared to 1 hpi for D109 (Fig. 2A) and D2004 (Fig. 2B) HIE lines. Similar to the VLP studies, only 20 mg/mL of 2′FL significantly inhibited GII.4 Sydney [P16] HuNoV replication at 24 hpi. In evaluating the effect of 2′FL on GII.4 Sydney [P16] HuNoV binding at 1 hpi, 20 mg/mL 2′FL significantly reduced binding in D2004 but not in D109 HIE. None of the 2′FL concentrations tested were cytotoxic to HIEs, as measured by the lactase dehydrogenase (LDH) assay.

**Fig 3:**
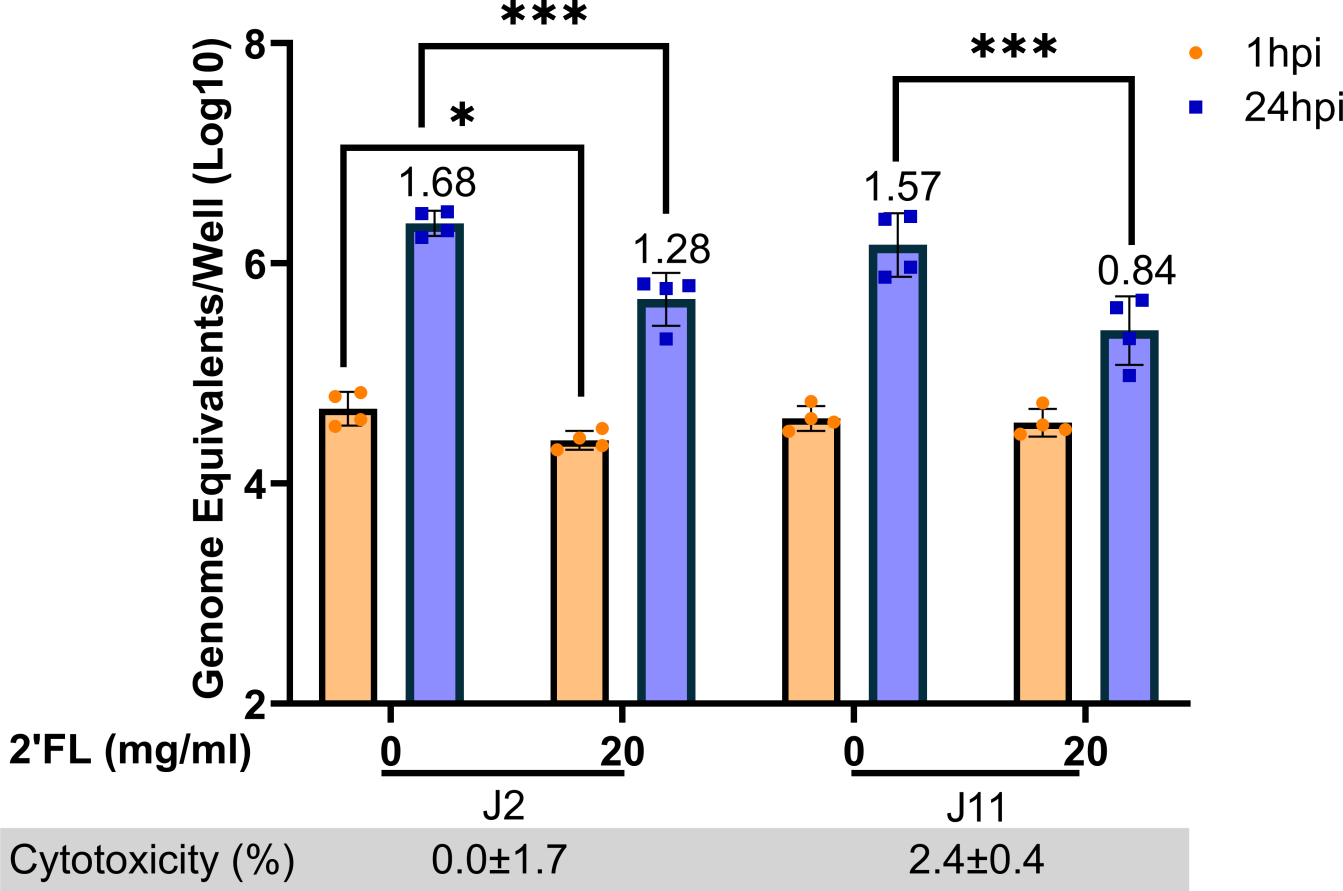
2′FL significantly reduces GII.4 Sydney [P16] HuNoV replication in adult jejunal HIE lines. 20 mg/mL of 2′FL was tested in two adult jejunal HIEs J2 and J11. GE/well was determined by RT-qPCR at 1 and 24 hpi. Numbers above the bars indicate log_10_ fold change comparing GE/well at 24 to 1 hpi. Cytotoxicity is represented in percentage below each graph. Data represented are means ± standard deviation (SD) from *n* = 2 independent experiments with two technical replicates per experiment. The P-values were calculated using analysis of variance, Sidak’s multiple comparisons test. *P ≤ 0.05, ***P ≤ 0.001.

To evaluate if the reduction in GII.4 Sydney [P16] replication with 2′FL could be seen in other intestinal segments, we next tested 2′FL in two adult jejunal HIE lines J2 and J11. Of note, we performed this and subsequent experiments only with 20 mg/mL of 2′FL since PGM-VLP assays and dose-response infectivity studies in duodenal HIEs showed significant results only at the highest concentration. Both in J2 and J11 HIEs (Fig. 3), GII.4 Sydney [P16] HuNoV showed ~1.5 log_10_ increase at 24 hpi compared to 1 hpi in the absence of 2′FL (0 mg/mL). Treatment with 20 mg/mL of 2′FL resulted in a significant reduction in GII.4 Sydney [P16] HuNoV replication for both lines. When comparing HuNoV replication in the absence of 2′FL at 24 hpi, there was a 0.4 log_10_ decrease in J2 and 0.7 log_10_ decrease in J11 HIE. Similar to the duodenal HIEs, 20 mg/mL of 2′FL also showed a significant decrease in GII.4 Sydney [P16] binding for one jejunal HIE line (J2) but not the other. 20 mg/mL of 2′FL was not cytotoxic in either J2 or J11 HIE.

**Fig 4:**
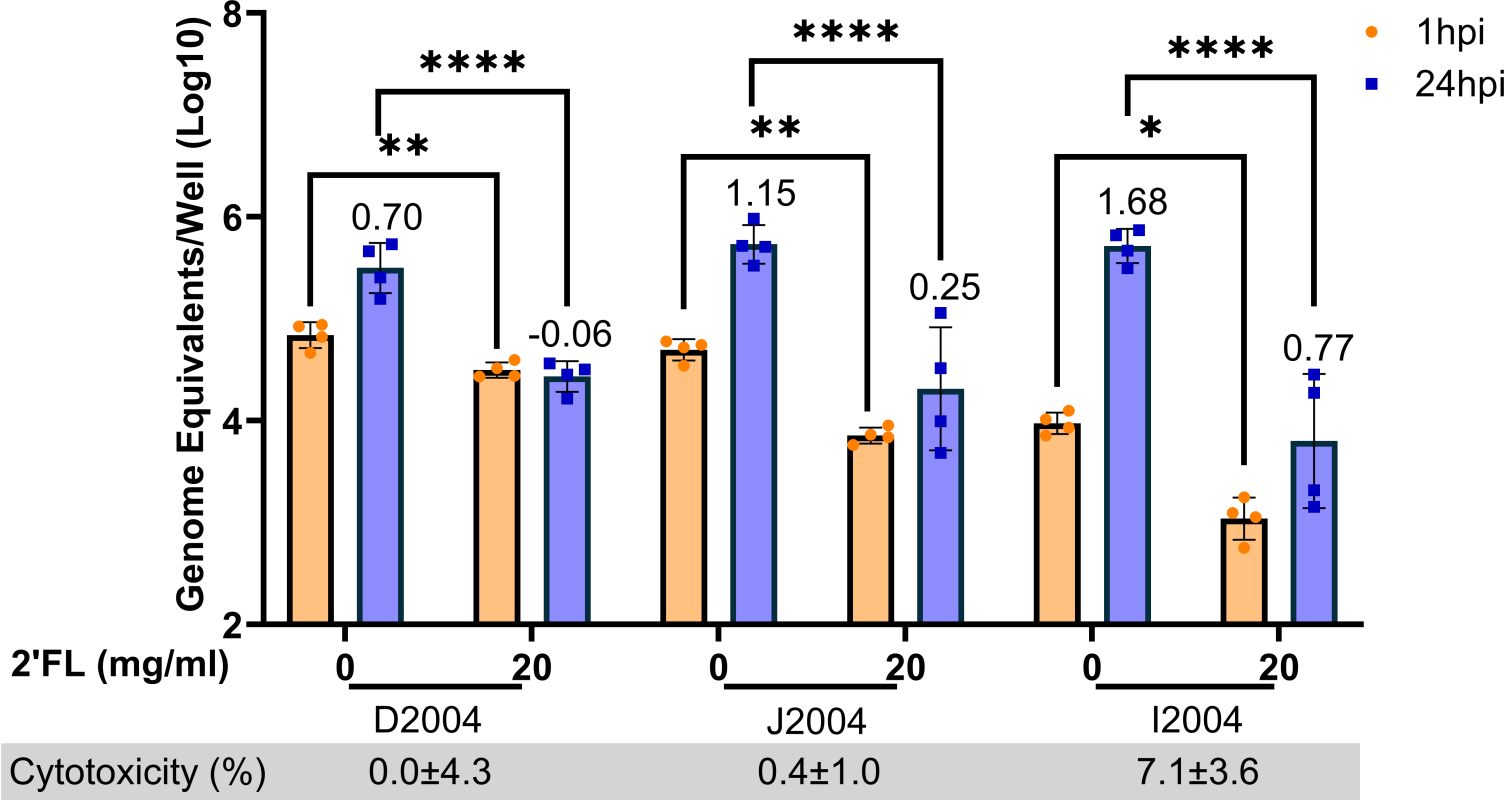
2′FL significantly reduces GII.4 Sydney [P16] HuNoV binding and replication in all segments from the same adult donor. Studies testing 20 mg/mL of 2′FL in duodenal (D2004), jejunal (J2004), and ileal (I2004) HIEs from an adult donor were carried out. GE/well was determined by RT-qPCR at 1 and 24 hpi. Numbers above the bars indicate log_10_ fold change comparing GE/well at 24 to 1 hpi. Cytotoxicity is represented in percentage below each graph. Data represented are means ± standard deviation (SD) from *n* = 2 independent experiments with two technical replicates per experiment. The P-values were calculated using analysis of variance, Sidak’s multiple comparisons test. *P ≤ 0.05, **P ≤ 0.01, ****P ≤ 0.0001.

Taken together, these data indicate that 20 mg/mL 2′FL significantly inhibits GII.4 Sydney [P16] HuNoV replication in duodenal and jejunal HIEs. However, the magnitude of replication and inhibition varied between the different HIE lines. Since all the HIEs tested thus far were derived from different adult donors, it is possible that some of these differences could be attributed to variability between donors. We, therefore, wanted to evaluate the effect of 2′FL in intestinal segments from the same donor. 20 mg/mL of 2′FL was tested in duodenal (D2004), jejunal (J2004) and ileal (I2004) HIEs from a single donor. Replication was highest in the ileum, as measured by fold increases in GE/well from 1 to 24 hpi, followed by jejunum, and then duodenum (Fig. 4). 20 mg/mL 2′FL significantly decreased both binding and replication of GII.4 Sydney [P16] HuNoV in all segments, with complete inhibition seen in the D2004 line. 2′FL was not cytotoxic in any of the segments.

**Fig 5:**
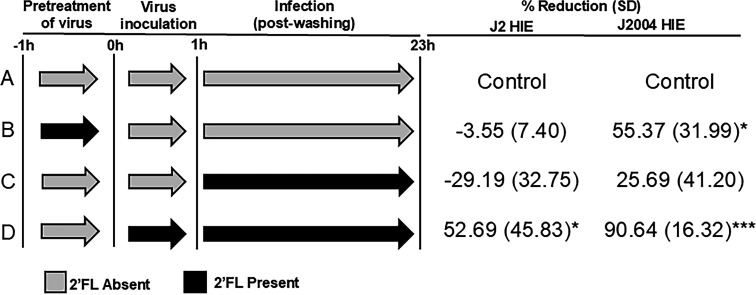
2′FL significantly inhibits GII.4 Sydney [P16] HuNoV replication in J2 and J2004 HIEs when present throughout the course of infection. A schematic representation of when 2′FL was added to the virus or the cells is shown on the left, with gray and black arrows indicating the absence and presence of 2′FL, respectively. The average % reduction in replication compared to no 2′FL treatment for J2 and J2004 HIEs is shown on the right. Data represented are means ± standard deviation (SD) from *n* = 2 independent experiments with two technical replicates per experiment. The *P*-values were calculated using analysis of variance, Sidak’s multiple comparisons test. *P ≤ 0.05, ***P ≤ 0.001.

In all experiments described thus far, 2′FL was added during the initial binding of virus to HIEs for 1 h and also present in the media for 24 hpi. Using two HIE lines (J2 and J2004) and no 2′FL treatment as control, we next evaluated whether infection of HIEs with GII.4 Sydney [P16] HuNoV pre-incubated with 2′FL or the addition of 2′FL to HIEs following onset of HuNoV infection could also inhibit virus replication (Fig. 5, conditions A–C). The standard infection condition used for the data presented in Fig. 2 to 4 was included as a positive control (Fig. 5, condition D). While HIE-line specific differences in results were observed for each treatment condition, the most significant reduction in replication for both lines was seen when 2′FL was present during the entire course of infection (Fig. 5, condition D). For the J2 HIE line, neither pre-treatment of virus nor addition of 2′FL following infection showed any inhibition in HuNoV replication. By contrast, GII.4 Sydney [P16] HuNoV replication was significantly reduced with pre-treatment of virus and also reduced with addition of 2′FL following inoculation in the J2004 HIE line. The replication of GII.4 Sydney [P16] HuNoV was higher in J2 compared to J2004 HIE (Fig. 3 and 4, respectively), raising the possibility that higher concentrations of 2′FL may be needed to achieve similar inhibition in J2 HIE as the J2004 line.

### 2′FL significantly reduces GII.4 human norovirus replication in pediatric duodenal but not infant jejunal HIE lines

**Fig 6:**
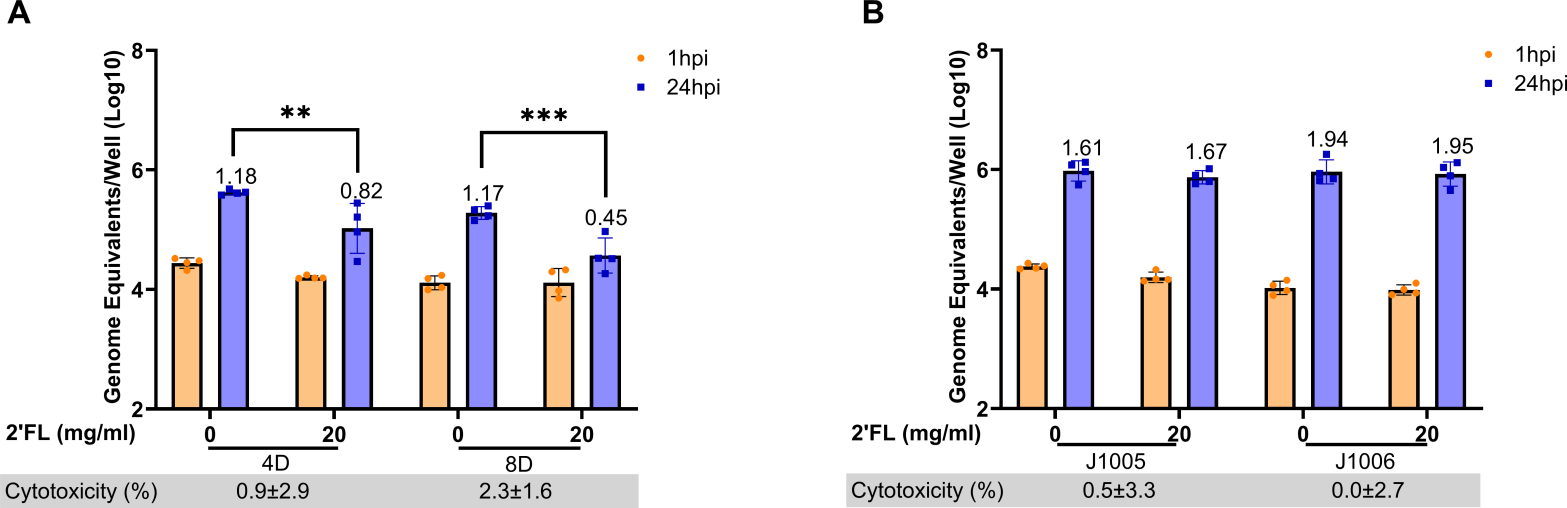
2′FL significantly reduces GII.4 Sydney [P16] HuNoV replication in pediatric duodenal but not infant jejunal HIE lines. 20 mg/mL of 2′FL was tested in (**A**) two pediatric duodenal HIEs (4D and 8D) and (**B**) two infant jejunal HIEs (J1005 and J1006). GEs per well were determined by RT-qPCR at 1 and 24 hpi. Numbers above the bars indicate log_10_ fold change comparing GEs at 24 to 1 hpi. Cytotoxicity is represented in percentage below each graph. Data represented are means ± standard deviation (SD) from *n* = 2 independent experiments with two technical replicates per experiment. The *P*-values were calculated using analysis of variance, Sidak’s multiple comparisons test. **P ≤ 0.01, ***P ≤ 0.001.

As 2′FL significantly reduced HuNoV replication in adult lines, we next wanted to determine if similar outcomes would be observed in pediatric and infant HIE lines. Infectivity studies were carried out in two pediatric duodenal lines (4D and 8D, Fig. 6A) and two infant jejunal lines (J1005 and J1006, Fig. 6B). Similar to adult HIEs, higher replication in the absence of 2′FL was seen in infant jejunal HIEs (1.8log_10_) compared to pediatric duodenal HIEs (1.2log_10_). 20 mg/mL 2′FL reduced HuNoV replication, but not binding, in the two pediatric duodenal HIE lines. Surprisingly, when 20 mg/mL 2′FL was tested in two infant jejunal HIE lines (J1005 and J1006), no reduction of HuNoV binding or replication was observed (Fig. 6B), suggesting that 2′FL is not acting as a decoy to block virus replication in these lines. 20 mg/mL 2′FL was not cytotoxic in both pediatric duodenal and infant jejunal lines.

### Infant jejunal HIEs express lower level of α1–2-fucosylated HBGAs

**Fig 7:**
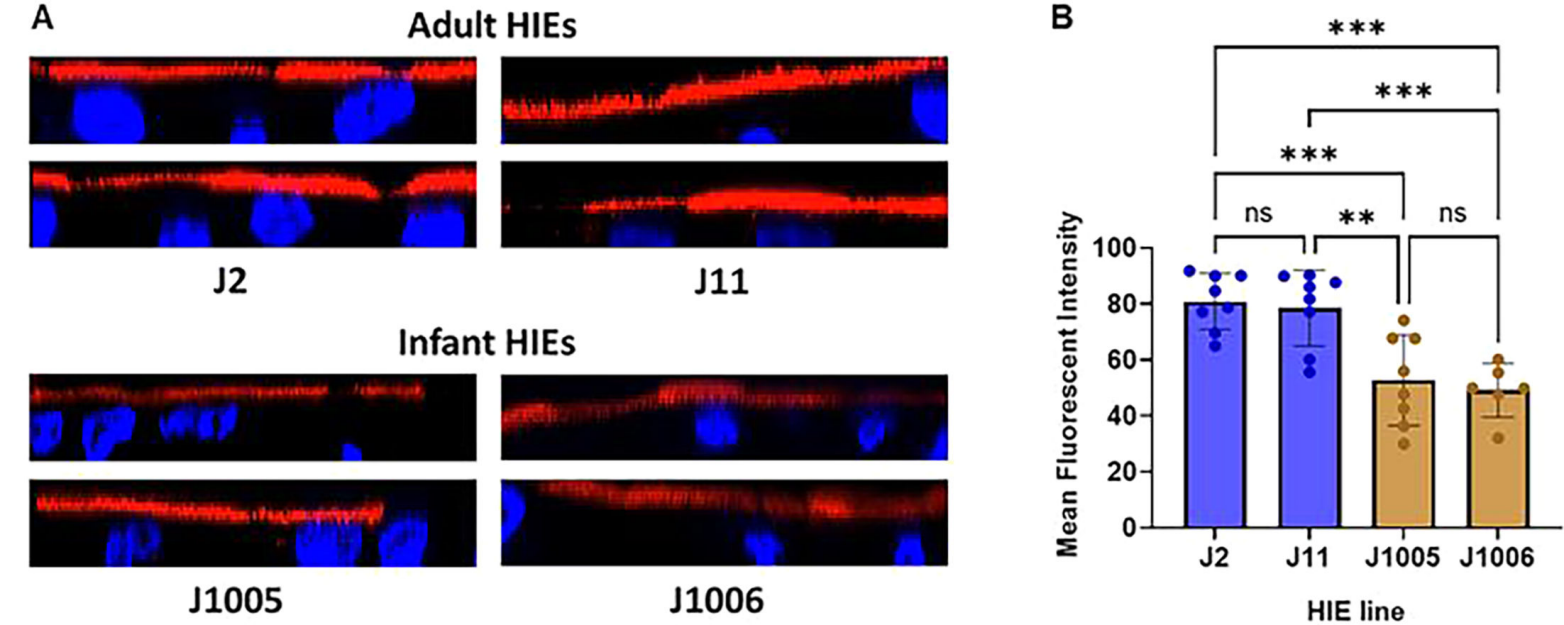
Level of HBGA expression is lower in infant jejunal HIE lines as compared to adult jejunal HIE lines. (**A**) Infant jejunal lines (J1005 and J1006) and adult jejunal lines (J2 and J11) stained with *Ulex europaeus* Agglutinin-1 (UEA-1) were imaged using confocal microscopy. Two representative images are shown per HIE line. (**B**) Fluorescence intensity was measured for each line using FIJI/ImageJ. Two to four fields per well were analyzed. Mean fluorescence data from five identical regions of interest (ROIs) per two to four fields were averaged. The *P*-values were calculated using analysis of variance, Holm–Sidak’s multiple comparisons test. **P ≤ 0.01, ***P ≤ 0.001. *N* = 2 independent experiments.

As 20 mg/mL 2′FL did not inhibit GII.4 Sydney [P16] replication in the infant jejunal lines but inhibited replication in the adult jejunal HIE lines, we wanted to evaluate if there was a lower expression of fucosylated HBGAs in the infant lines. We compared the expression of α1–2-fucosylated glycans between the adult jejunal lines (J2 and J11) and infant jejunal lines (J1005 and J1006) by staining the HIEs with *Ulex europaeus* Agglutinin-1 (UEA-1, Fig. 7A) (24). Significantly lower fluorescent intensity was observed in the infant jejunal lines compared to the adult jejunal lines (Fig. 7B), suggesting the possibility of additional binding factors in the infant HIE lines other than α1–2-fucosylated HBGAs. There is no significant difference in fluorescent intensity between the two adult jejunal lines or between the two infant jejunal lines (Fig. 7B).

## DISCUSSION

HMOs are known to act as decoy receptors for multiple enteric pathogens (25, 26). Previous studies have demonstrated that milk from secretor mothers, who produce α1–2-fucosylated HMOs, could block the binding of prototype Norwalk virus (GI.1) VLPs to intestinal tissues, H type 1 HBGA, and saliva (27–29). Subsequent studies showed that 2′FL can block the binding of GI.1, GII.4, GII.10, and GII.17 VLPs to PGM and saliva samples from multiple donors (19, 21, 22). X-ray crystallography studies revealed that 2′FL binding occurred at the HBGA binding pockets suggesting that 2′FL can act as a decoy receptor for multiple HuNoV strains. However, phenotypic data on 2′FL interactions with the globally dominant GII.4 genotype have been variable. Two previous studies using VLPs from the VA387 GII.4 strain suggested weak binding to 2′FL and the need for higher-molecular weight glycoconjugates for inhibiting carbohydrate ligand interactions (30, 31). 2′FL at concentrations as high as 24 mg/mL did not inhibit GII.4 Sydney [P16] replication in zebrafish larvae, although inhibition of binding to A-type saliva was seen (32). By contrast, structural studies suggest that the protruding domain of the GII.4 Sydney capsid protein binds 2′FL and HBGAs in the same pocket (20). Corresponding to this data, a recent study showed that crushed 2′FL tablets and 2′FL powder inhibited GII.4 VLP binding to A-type saliva (22). In the present study, we evaluated the effect of 2′FL on the infectivity of a recently circulating GII.4 Sydney [P16] HuNoV strain in HIEs. These nontransformed cultures serve as a physiologically relevant model system of the small intestinal epithelium and retain intestinal segment specificity as well as donor phenotypic characteristics. We discovered that 2′FL inhibits GII.4 Sydney [P16] HuNoV replication in multiple adult HIE lines without cytotoxicity (summarized in Table 2), and, thus, has the potential to be developed as a therapeutic for HuNoV gastroenteritis.

**TABLE 2:** Summary of the effect of 2′FL on GII.4 Sydney [P16] HuNoV replication^*a*^

HIE	Segment	Age	Average log_10_ fold increase in the absence of 2′FL	Average log_10_ fold increase with 20 mg/mL 2′FL (% reduction)
D109	Duodenal	44 years	1.15	0.34 (70.4%)
D2004	Duodenal	25 years	0.82	0.11 (86.6%)
J2	Jejunal	52 years	1.68	1.28 (23.8%)
J11	Jejunal	52 years	1.57	0.84 (46.5%)
J2004	Jejunal	25 years	1.15	0.25 (78.3%)
I2004	Ileal	25 years	1.68	0.77 (54.2%)
4D	Duodenal	2 years	1.18	0.82 (30.5%)
8D	Duodenal	5 years	1.17	0.45 (61.5%)
J1005	Jejunal	10 weeks	1.61	1.67 (0%)
J1006	Jejunal	12 weeks	1.94	1.95 (0%)

^*a*^ List of HIE lines, in which the segment of origin and their respective age are shown. Average log_10_ fold increase in the absence of 2′FL and with 20 mg/mL 2′FL (percentage reduction) is shown.

The concentration of 2′FL that inhibits GII.4 Sydney [P16] replication in HIEs is consistent with biochemical studies with GI.1, GII.4, GII.17, and GII.10 VLPs where the IC_50_ was calculated to be between 5 and 20 g/L (19, 21, 22). While these concentrations are substantially higher than average concentrations in human milk, the safety profile of higher concentrations of 2′FL has been evaluated previously. A preclinical study in rats showed that oral administration of 2′FL up to 5,000 mg per kilogram of body weight per day for over 90 days was not associated with any adverse effects based on clinical observations and histopathology, body weight gain, and food consumption (33). A randomized, double-blind, placebo-controlled, oral supplementation study of 2′FL in 100 healthy adults showed that up to 20 g/day for about 12 days was safe and well tolerated (34). Microbiome composition analysis using 16S rRNA sequencing showed that HMO supplementation resulted in changes in the gut microbiota with increases in the relative abundance of Actinobacteria and *Bifidobacterium* and a reduction in the relative abundance of Firmicutes and Proteobacteria. Chemical, chemo-enzymatic, and enzymatic strategies to produce 2′FL have been described and include strategies for kilogram-scale synthesis (35). Multiple 2′FL manufacturers, including the one for the 2′FL used in this study, have received “no questions” letters from the US Food and Drug Administration regarding the generally recognized as safe notices for use of their HMO (36). Also, the European Food Safety Authority has published positive assessment opinions for use of 2′FL in food supplements. Infant formula supplemented with 2′FL is well tolerated in healthy-term infants and supports age-appropriate growth (37–39). Additional health benefits of 2′FL have been described in various studies. Unbiased metabolomic analyses and short chain fatty acid production were evaluated in bioreactors seeded with fecal samples from six adults and six children (6 year old) that were supplemented with 0.5–1 g per day equivalent of 2′FL; these studies demonstrated significant increases in acetate and propionate production, as well as aromatic lactic acids linked to immune function (40). 2′FL was also associated with a significant reduction in fluorescein isothiocyanate-dextran permeability in Caco2 cells and upregulation of tight junction proteins like Claudin-5 in colon-on-chip models under microfluidic conditions (41). Taken together, these data provide a promising outlook to regulatory pathways for clinical testing of 2′FL as an inhibitor for HuNoVs.

A critical observation in our study was that the inhibition of HuNoV replication varied by donor, intestinal segment, and age. The relative contribution of each of these factors remains to be elucidated. However, the availability of HIEs from all segments of the small intestine from a single donor allowed us to confirm that 2′FL can inhibit GII.4 Sydney [P16] replication across multiple segments. While the level of inhibition varied, with complete inhibition in duodenal HIEs to approximately 50% inhibition in ileal HIEs, it is to be noted that the magnitude of replication also varied, with the highest replication seen in ileal HIEs despite using 100 TCID_50_ of virus in all lines. The complete lack of inhibition in infant jejunal HIEs is particularly striking. We previously demonstrated significant transcriptional, morphological, and functional differences between the adult and infant jejunal HIEs used in this study (42). Of relevance to HMOs, the expression of lactase (β-galactosidase) was significantly higher in infant HIEs. However, previous studies have postulated that despite significant lactase presence, the upper small intestine of piglets and infants do not cleave HMOs (43). We evaluated differences in the HBGA expression between infant and adult jejunal HIEs. The significantly lower expression of α1–2-fucosylated glycans on infant jejunal HIEs in comparison to adult lines suggests the possibility of additional cellular attachment factors on infant lines, which allow viral infection and replication to occur despite the decoy activity of 2′FL.

Future studies can be performed to address some limitations of this work. First, additional mechanistic studies in several HIE lines are required to determine the relative contributions of the 2′FL decoy receptor activity and host responses to 2′FL treatment to observe antiviral effects. This was evident in two HIE lines where we observed that the magnitude of inhibition was greater when 2′FL was present throughout the course of infection than when 2′FL was used to pre-treat the virus or when 2′FL was added after the onset of infection. Second, while some lines show complete inhibition of GII.4 Sydney [P16] replication, the range of effects is large. Concentrations of 2′FL beyond 20 mg/mL were not tested in this study, and it is possible that higher concentrations may lead to more inhibition of HuNoV replication. While this could be assessed in future studies, longer chain fucosylated HMOs like lacto-N-fucopentaose I or combinations of 2′FL with the other HMOs, such as 3-fucosyllactose (3FL), can be tested as additional approaches to determine if consistent reduction in replication can be achieved with lower concentration of HMOs across different donors and segments. A recent structural study with nanobodies also demonstrated increased potency when used in combination with 2′FL (44). Such combination strategies could also be evaluated in future studies for effects on virus replication. Third, to evaluate the broad applicability of 2′FL or modified glycoconjugates, the effect on replication of additional HuNoV strains and in additional HIE lines in each age category/segment needs to be evaluated. Whether early and late passages of HIEs show differences in antiviral effects can also be determined in such studies. Finally, assessment of 2’FL effects in HIEs from infants, toddlers, and older children will allow us to determine whether there are developmentally regulated differences between receptor/co-receptor expression for HuNoVs.

We recently standardized a pipeline for evaluation of antivirals against HuNoVs using HIEs and applied this pipeline to evaluate nitazoxanide, an anti-parasitic drug that is anecdotally used for the treatment of chronic HuNoV infections in immunocompromised patients (23). In that study and in others evaluating neutralizing antibodies, we used a 90% reduction in virus yield (1log_10_) to evaluate inhibition of virus replication. This cutoff was shown to yield less variability between results in virus inhibition in HIEs (23, 45–47). The present study demonstrates the utility of this pipeline to preclinically evaluate compounds based on the known biology of HuNoVs and establishes the potential for 2′FL to be developed as a therapeutic for adults. This finding is significant because previous studies have focused primarily on structural interactions and carbohydrate ligand blocking and did not demonstrate inhibition of virus replication. Further testing in clinical studies, such as a controlled human infection model, will be required to determine the dosage and duration of 2′FL treatment that results in virus clearance. Despite the high burden of disease, there are currently no approved antivirals or therapeutics for treating HuNoV infections, and a 2′FL-based oral therapeutic could have prophylactic applications in settings of high risk for outbreaks, such as cruise ships, or in treatment for acute or chronic infections.

## MATERIALS AND METHODS

### VLP, virus, and 2′FL

GII.4 Sydney 2012 VLPs were used for the initial screening assay to evaluate whether 2′FL blocks the binding to PGM. VLPs were produced in a baculovirus system using open reading frame 2 (ORF2) + ORF3 + untranslated region (UTR) sequences (48). A GII.4 Sydney [P16] strain (strain BCM 16–16, stock titer 4.26 × 10^6^ GE/µL) was used for all infectivity experiments. 2′-Fucosyllactose, produced in bioengineered microbes, was generously provided in-kind by Jennewein GmbH, Germany, which was later acquired by Chr Hansen, Denmark, now part of Novonesis.

### Human intestinal enteroids

Secretor-positive HIE lines from different intestinal segments and donors of different ages were used in this study. These include two adult duodenal lines (D109, D2004), three adult jejunal lines (J2, J11, J2004), and one adult ileal line (I2004). Of the adult lines, D2004, J2004, and I2004 were obtained from a single donor (49). In addition to HIE lines from adults, two pediatric duodenal lines (4D, 8D) and two infant jejunal (J1005, J1006) were included in this study. The ages of the HIE donors are listed in Table 1. HIE cultures were generated at the Texas Medical Center Digestive Diseases Center Gastrointestinal Experimental Model Systems Core from intestinal crypts isolated from patient biopsies or surgical tissues. The samples were collected under IRB-approved protocols at Baylor College of Medicine and the University of Texas Health Science Center at Houston, as described previously (42, 50). The pediatric duodenal lines (4D and 8D) were originally established by Dr. Mark Donowitz at Johns Hopkins University Medical School and obtained through a Material Transfers Agreement.

### HBGA blocking assays

An assay used to evaluate serum HBGA blocking activity was modified to evaluate the blocking activity of 2′FL (51). Briefly, 96-well polystyrene flat-bottom plates (Greiner Bio-One, 655001) were coated with 3 µg/mL PGM diluted in 0.01 M phosphate buffer saline (PBS) overnight at 4°C on a rocking platform. Following incubation, 1% non-fat dry milk in 100 mM sodium phosphate buffer (PB) and pH 6.1 was added to the PGM-coated plates and incubated for 2 h at room temperature protected from light. Meanwhile, two-fold dilutions of 2′FL ranging from 1.25 to 20 mg/mL were incubated with 2.5 µg/mL GII.4 Sydney 2012 VLPs in tissue-culture treated round-bottom plates (Corning, 3799) at 4°C on a rocking platform for an hour. As the positive control, 2.5 µg/mL of GII.4 Sydney 2012 VLPs were diluted with PB buffer. Following incubation, the PGM coated plates were washed five times with cold PB buffer. The 2′FL-VLP solutions were transferred to the PGM coated plates and incubated at 4°C for 2 h protected from light. Following incubation, the plates were washed five times with cold PB buffer. An in-house guinea pig anti-GII.4 Sydney primary antibody (1:3,000) was added to the wells. The plate was incubated at 4°C for 1 h protected from light. The plates were washed five times with cold PB buffer, and goat anti-guinea pig secondary antibody conjugated with HRP (1:5000, Sigma, A7289) was added to the wells. The plates were incubated at 4°C for 1 h protected from light. After washing, TMB (3,3′,5,5′-tetramethylbenzidine) substrate (KPL, 5120-0047) was added to all the wells for 10 min protected from light. 1 M phosphoric acid was used as the stop solution, and the absorbance was measured at 450 nM using a microplate reader (Spectramax). The VLP binding assays were performed three times, with three technical replicate wells for each condition in an experiment.

### Human norovirus infectivity

Three-dimensional HIE cultures were obtained from the Gastrointestinal Experimental Model Systems Core (GEMS) of the Texas Medical Center Digestive Diseases Center (TMC DDC) and plated as monolayers on 96-well plates, as described previously, using commercially available Intesticult Organoid Growth Medium (OGM) proliferation and differentiation media (52, 53). The GE/TCID_50_ was determined for each HIE line as described previously so that a standard dose of virus could be used across different HIE lines (23). 2′FL was diluted in OGM differentiation media with 500 µM sodium glycochenodeoxycholate (GCDCA; Sigma, G0759) and added to 5-day differentiated HIE monolayers on a 96-well plate (Corning, 3595) with 100 TCID_50_ of virus. One hundred TCID_50_ virus in the absence of 2′FL was used to determine baseline infectivity in the absence of treatment. Following incubation at 37°C for 1 h, the HIE monolayers were washed three times with complete media without growth factors (CMGF−). OGM differentiation media with 500 µM GCDCA was added to the control wells. 2′FL diluted in OGM differentiation media with 500 µM GCDCA was added to the treatment wells. The samples were incubated for a further 23 h at 37°C.

In addition to these studies, we also performed infectivity assays where either the virus was pre-treated with 2′FL, or 2’FL was added after the onset of infection. For pretreating the virus with 2′FL, a 100 TCID_50_ of virus was mixed with 20 mg/mL 2′FL and incubated at 4°C for an hour. The virus–2′FL mixture was then added to HIEs for 1 h at 37°C. After washing the monolayers three times with CMGF−, 20 mg/mL 2′FL was added, and the cells were incubated for a further 23 h at 37°C. For evaluating the effect of 2′FL after viral inoculation, 100 TCID_50_ virus was added to the cells for an hour for binding. Then, the HIE monolayers were washed three times with CMGF−, and 20 mg/mL 2′FL was added to the HIEs. The cells were incubated for 23 h at 37°C. A schematic representation of the different treatment conditions is shown in Fig. 5.

In all experiments, total RNA was extracted using a KingFisher Flex machine (Thermo Fisher) and a MagMax-96 Viral RNA Isolated Kit (Applied Biosystems), as described previously (52). RT-qPCR (Applied Biosystems) was carried out for the extracted RNA samples, and viral replication was quantitated relative to a standard curve. GE/µL measured at 1 and 24 hpi was used to estimate input virus and replication, respectively. Each infectivity experiment was performed twice with two technical replicate wells for each condition within an experiment. RT-qPCR assays were carried out using three technical replicates for each HIE well.

### Cytotoxicity assessment

Cytotoxicity assays were carried out in tandem with the viral infectivity assays using the CytoTox 96 Non-radioactive Cytotoxicity Assay (Promega, G1780). The assay was carried out according to the manufacturer’s instructions with some modifications wherein supernatants were diluted in media to achieve optical density (OD) values in the linear range of the assay (54). OD values were taken using a microplate reader at 490 nM (Spectramax), and percent cytotoxicity was calculated for each sample.

### UEA-1 staining

Five-day differentiated HIE monolayers plated on tissue culture-treated slides (Ibidi, 80826) were fixed with 4% paraformaldehyde (Electron Microscopy Sciences, 15710S) for 25 min at room temperature. The cells were incubated overnight at 4°C with rhodamine-conjugated UEA-1 (Vector Laboratories, RL-1062–2) diluted 1:200 in 5% bovine serum albumin in 0.01 M PBS + 0.1% Triton (24). The cells were washed with 0.01 M PBS + 0.1% Triton three times (10 min incubations), and nuclei were stained with a NucBlue Fixed Cell Stain ReadyProbes reagent (Invitrogen, R37606) diluted in 0.01 M PBS for 5 min. Orthogonal sections of the cells were imaged using a Zeiss confocal microscope (Laser Scanning Microscope LSM 980) using ZEISS ZEN 3.5 (blue edition) software. The images were further processed and analyzed using ImageJ2/FIJI. For quantifying fluorescence intensity, two to four fields per well were analyzed. Mean fluorescence data from five identical regions of interest (ROIs) per field were collected. The experiments were performed twice with two technical replicate wells in each experiment for each HIE line.

### Statistical analysis

GraphPad Prism 9.5.1 was used for all statistical analyses. For the PGM-VLP assays, Student’s *t*-test was used to compare the 2′FL concentrations to the control. For the infectivity assays, comparison between 1 and 24 hpi groups in the presence and absence of 2′FL was performed using a two-way analysis of variance (ANOVA) and Sidak’s post-hoc multiple comparisons analyses. For comparing fluorescent intensities of UEA-1 staining in the immunofluorescence assays between the different lines, a one-way ANOVA was performed using Holm–Sidak’s multiple comparisons test for post-hoc analyses. Error bars denote standard deviation (SD) for all graphs.

## Data Availability

Data that support the findings of this study are available from the corresponding author on request.
